# Extracellular Cold-Inducible RNA-Binding Protein and Hemorrhagic Shock: Mechanisms and Therapeutics

**DOI:** 10.3390/biomedicines13010012

**Published:** 2024-12-25

**Authors:** Naureen Rashid, Zhijian Hu, Asha Jacob, Ping Wang

**Affiliations:** 1 Center for Immunology and Inflammation, Feinstein Institutes for Medical Research, Manhasset, NY 11030, USA; nrashid1@northwell.edu (N.R.); zhu1@northwell.edu (Z.H.); 2 Department of Molecular Medicine, Zucker School of Medicine at Hofstra/Northwell, Manhasset, NY 11030, USA; 3 Department of Surgery, Zucker School of Medicine at Hofstra/Northwell, Manhasset, NY 11030, USA

**Keywords:** extracellular CIRP, trauma hemorrhage, ischemia and reperfusion, organ damage, inflammation

## Abstract

Hemorrhagic shock is a type of hypovolemic shock and a significant cause of trauma-related death worldwide. The innate immune system has been implicated as a key mediator in developing severe complications after shock. Inflammation from the innate immune system begins at the time of initial insult; however, its activation is exaggerated, resulting in early and late-stage complications. Hypoxia and hypoperfusion lead to the release of molecules that act as danger signals known as damage-associated molecular patterns (DAMPs). DAMPs continue to circulate after shock, resulting in excess inflammation and tissue damage. We recently discovered that cold-inducible RNA-binding protein released into the extracellular space acts as a DAMP. During hemorrhagic shock, hypoperfusion leads to cell necrosis and the release of CIRP into circulation, triggering both systemic inflammation and local tissue damage. In this review, we discuss extracellular cold-inducible RNA-binding protein (eCIRP)’s role in sterile inflammation, as well as its various mechanisms of action. We also share our more newly developed anti-eCIRP agents with the eventual goal of producing drug therapies to mitigate organ damage, reduce mortality, and improve patient outcomes related to hemorrhagic shock. Finally, we suggest that future preclinical studies are required to develop the listed therapeutics for hemorrhagic shock and related conditions. In addition, we emphasize on the challenges to the translational phase and caution that the therapy should allow the immune system to continue to function well against secondary infections during hospitalization.

## 1. Introduction

Hemorrhagic shock is one of the leading causes of death worldwide, contributing to approximately 1.9 million deaths per year, of which 1.5 million are related to trauma [1]. The mortality rate from uncontrolled hemorrhage in trauma is staggeringly high, reaching up to 40% in some cases [2,3]. In addition to active hemorrhage and traumatic insult, the procedures to mitigate blood loss also carry the risk of worsening shock. With endovascular treatments on the rise, increased occlusion time along with inadvertent vessel damage can contribute to increased hemorrhage and hypoxemia [4]. Hemorrhagic shock is a type of hypovolemic shock in which severe blood loss leads to the inadequate perfusion of the tissues and compensatory shunting of blood to vital organs, such as the brain and heart. Vasoconstriction in response to reduced blood pressure exacerbates poor perfusion and worsens ischemia. Prolonged ischemia from the depleted intravascular volume eventually results in global ischemia [1]. As oxygenated blood fails to reach tissues in adequate amounts, the cells convert from aerobic to anaerobic metabolism. Lactic acid, as a byproduct of anaerobic metabolism, accumulates to create an acidic environment, causing local tissue damage. The acidotic environment coupled with altered ion balance results in the overactivation of proteases and increased membrane permeability. These processes compromise cellular integrity, leading to apoptosis, necrosis, autophagy and necroptosis [5].

Early mortality in traumatic injury is due to the “lethal triad” which consists of acidosis, hypothermia, and coagulopathy. These morbid physiological aberrations result in uncontrolled bleeding and an inability to recover from shock. Late mortality, however, is usually due to multi-organ failure through a combination of systemic inflammatory response syndrome (SIRS) and secondary immunosuppression [6]. Following resuscitation, patients develop severe complications including ischemia–reperfusion injury, delayed infections, immune dysfunction, and acute lung injury [7,8,9,10]. Late-stage complications often arise from reperfusion injury and prolonged immune dysfunction. Reperfusion gives rise to further local inflammation as well as the generation of reactive oxygen species, calcium overload, secondary neutrophil infiltration, and a prothrombotic state [5]. It is often these late complications that result in death, and many clinical researchers have chosen to focus on 28-day mortality. Currently, therapy for hemorrhagic shock is limited to damage control by localizing and controlling the source of bleeding along with closely monitoring resuscitation. Methods for optimal resuscitation have historically been in flux, with crystalloid falling out of favor more recently and in favor of colloid resuscitation with blood products. Excessive crystalloid resuscitation gives rise to dilutional coagulopathy, worsening prognosis. Resuscitation with blood products is the mainstay of treatment. The PAMPer clinical trial from 2018, funded by the U.S. Army, demonstrated reduced 30-day morbidity (23.2% vs. 33.0%) when patients were transfused with thawed plasma at the prehospital phase [11]. Despite advances in surgical technique and ICU care, molecular treatment options remain scarce. The most promising clinical trial data come from the CRASH-3 and PATCH trauma trials regarding tranexamic acid (TXA). In the CRASH-3 trial from 2019, patients with traumatic brain injury and subsequent intracranial bleeding risk of death from head injury decreased from 19.8% to 18.5% when patients were treated with TXA within 3 h of injury. https://www.thelancet.com/journals/lancet/article/PIIS0140-6736(19)32233-0/fulltext, accessed on 9 November 2019. In the 2023 PATCH trauma trial, patients with severe traumatic injury who had a high risk of trauma-induced coagulopathy were divided into TXA and placebo groups. The 28-day mortality was reduced from 21.8% in the placebo group to 17.3% in the TXA group [12]. Alternatively, in the CRYOSTAT-2 clinical trail from 2023, patients were divided into standard care vs. treatment with high-dose cryoprecipitate groups. In this study as well, the main target was treating coagulopathy; however, unlike the TXA-related trials, there was no difference in 28-day mortality between the standard and treatment groups [13]. Even the data on TXA, while statistically significant, did not show a substantial difference in outcomes, and mortality remains high. While these trials guide current trauma management, clinicians remain skeptical. As coagulopathy treatment has not provided significant improvement in care, researchers have turned their sights towards other cellular processes.

Increasing evidence points to the innate immune system as a promising target for therapeutic intervention. The innate immune system is modulated by pattern recognition receptors (PRRs). These receptors are expressed on the host’s innate immune cells and detect specific molecular patterns, resulting in the release of pro-inflammatory cytokines. The molecular structures that trigger this response include both surface markers on pathogens, such as lipopolysaccharides (LPS), and endogenous molecules released from apoptosis or cell death. The exogenous molecules from pathogens are called pathogen-associated molecular patterns (PAMPs), while the endogenous molecules released during cell death act as danger signals and are known as damage-associated molecular patterns (DAMPs) [14]. During hemorrhagic shock, injuries from ischemia and reperfusion result in cell necrosis and the release of DAMPs. These DAMPs are the basis of sterile inflammation, and its resultant inflammatory cascade is meant to protect against injury. However, the massive release of DAMPs in necrotic tissue results in a hyperactivated state and a positive feedback loop with uncontrolled inflammation [6]. Examples of DAMPs include: extracellular cold-inducible RNA-binding protein (eCIRP) [15], high mobility group box 1 protein (HMGB1) [16,17,18], S100 proteins, heat shock proteins (HSPs), uric acid, histones, and mitochondrial DNA (mtDNA) [19,20,21,22].

In order to assess the early inflammatory response and subsequent multiple organ dysfunction syndrome (MODS), a prospective observational study of 18 severely polytraumatized patients was carried out in Hospital General Universitario, Madrid, Spain. Patients with MODS were found to have higher levels of TNF-α and IL-6 early in the hospital admission along with a slower decrease in heat shock protein 70 kDa protein 1 (HSPA1A), suggesting its potential as a prognostic marker. Anti-Hsp70 levels were decreased at all time points after trauma, suggesting an immunosuppressive state [23]. HMGB1 has been studied extensively in the past and has long been known to have a deleterious effect on organ damage in hemorrhagic shock, whereas neutralizing its effects ameliorates injury [24]. A 2023 retrospective clinical study revealed that HMGB1 and histone 3 (H3) were elevated in patients who sustained severe traumatic injury 6 h and 12 h after admission. Increased HMGB1 at admission and increased H3 at 6 h post-admission were associated with fatal outcomes [25]. In addition, a 2018 study demonstrated that patients with elevated mtDNA 2 h after injury had a higher likelihood of developing multiple organ dysfunction syndrome. A higher injury severity score (ISS) was associated with higher mtDNA levels in peripheral blood. Animal studies revealed that the use of scavenging molecules to remove mtDNA from circulation had a protective effect against severe organ damage when in shock [26]. A systemic review of cell-free DNA of trauma patients in the ICU, with a total of 14 studies and inclusive of 904 patients, demonstrated a significant correlation between higher values of cell-free DNA and higher mortality [27]. As prognostic markers, these DAMPs can help guide treatment decisions by surgeons, including whether to proceed with damage control surgery or continue to monitor patients in the ICU. Markers for an immunosuppressed state can help clinicians decide at what point to supplement treatment with hydrocortisone, a practice that is not yet agreed upon by trauma surgery groups.

DAMPs and fibrinolytic markers are more strongly associated with poor outcomes while coagulation markers have no association, suggesting that DAMPs may play a key role in increasing morbidity and mortality after severe trauma. DAMPs, as both therapeutic targets and diagnostic markers, are currently studied as targets in other sterile inflammatory processes as well including transplantation, cancer, and autoimmune diseases. Although the research has not yet reached the clinical stage, it is nonetheless a promising avenue for treatment. Although there is a myriad of articles of preclinical studies on DAMPs in sterile inflammation, in this review, we will only focus on eCIRP, a relatively recently identified DAMP, but also on its emerging role in hemorrhagic shock. Therefore, the objective of this review article is to briefly introduce the pathophysiology of hemorrhagic shock, to provide an extensive review on eCIRP, and to describe its mechanisms of action as well as targeting it as a potential therapeutic for hemorrhagic shock.

## 2. Cold-Inducible RNA-Binding Protein

Cold-inducible RNA-binding protein (CIRP) was the first cold shock protein found in mammals during the late 90s [28]. It is an 18 kDa RNA-binding protein consisting of an RNA-binding amino-terminal and a glycine rich carboxy-terminal. Additionally, it has been mapped to chromosome 19p13.3 in humans. The amino acid sequence of human CIRP has 95.3% similarity to mouse CIRP, which makes it an excellent molecule for in vivo studies on inflammation [29]. On its initial discovery, CIRP expression was found to increase when temperature of the cellular environment decreased from 37 °C to 32 °C [30]. Since then, other stressors have been identified in playing a role in increased CIRP expression including UV radiation, hypoxia, heat shock, osmotic pressure, and endoplasmic reticulum stress [31,32,33]. Intracellular CIRP has a regulatory function in various cellular processes including DNA repair, immune modulation, redox signaling, cell proliferation, and apoptosis. Under physiological conditions, CIRP is constitutively expressed at a low level in the nucleus where it regulates RNA transcription and processing [34]. CIRP plays a crucial role in regulating DNA double-strand break repair and maintaining genomic stability [35]. In immune cells, CIRP serves as a mediator for activating the NOD-like receptor family, pyrin domain containing 3 (NLRP3) inflammasome and processing, and the secretion of IL-1β [36]. Intracellular CIRP also plays a role against oxidative stress in cells. It also exerts an overall antiapoptotic effect and promotes cell proliferation in certain malignancies [37,38,39].

When the inflammatory cascade results in the disruption of cellular membrane due to programmed cell death and necrosis, intracellular CIRP travels to the extracellular space to become extracellular CIRP (eCIRP). Additionally, CIRP can be actively transported to the extracellular space via lysosomal exocytosis [15]. In contrast to intracellular CIRP, eCIRP acts as a new DAMP [15,40,41]. eCIRP acts on various cells and tissues to induce inflammation, tissue damage, and cell death. The released eCIRP promotes the release of pro-inflammatory cytokines such as TNF-α, IL-1β, and IL-6. It also promotes the release and/or attenuates the function of other DAMPs such as HMGB1 [15], heat shock proteins [42], uric acid [36], and mitochondrial DNA [43]. eCIRP has been implicated in several inflammatory diseases including rheumatoid arthritis, osteoarthritis, chronic obstructive pulmonary disease, and idiopathic pulmonary fibrosis [29,44,45]. Studies have demonstrated its involvement in neuroinflammation, lung injury, and sepsis.

## 3. eCIRP and Sterile Inflammation

### 3.1. eCIRP and Hemorrhagic Shock

The potential involvement of eCIRP in hemorrhagic shock was first elucidated by us in 2013 [15]. The presence of eCIRP in sera from patients admitted to surgical intensive care units was evaluated. Five females and five males with an average age of 71 years, with APACHEII scores that ranged from 13 to 25, showed the presence of eCIRP in sera. The average blood collection time was 43 h after the onset of shock, which was defined by <90 mm Hg due to either active hemorrhage or a traumatic insult. In serum from healthy volunteers, eCIRP was barely detected. These results were replicated in vivo using a rat model of hemorrhagic shock. Male Sprague Dawley rats (*n* = 4–6) were anesthetized, and the femoral artery and veins were cannulated. The animals were bled to reach a mean arterial pressure (MAP) of 25–30 mm Hg, which was then maintained for 90 min. Following the shock period, animals were then resuscitated using lactated Ringer’s solution with two times the bleed-out volume over a period of 60 min. Using this method, eCIRP levels were additionally noted to be elevated in serum as early as 3 h after ischemia and resuscitation. eCIRP protein levels were elevated at 150 and 420 min in the liver and heart, respectively, while mRNA levels were significantly elevated by 2- to 4-fold at 240 min post-shock. In vitro experiments using RAW 264.7 cells demonstrated eCIRP release from macrophages exposed to hypoxia, while treatment with recombinant CIRP resulted in elevated levels of TNF-α, IL-6, and HMGB1 levels (*n* = 3). The increase in inflammatory molecules was attenuated when eCIRP was inhibited using a neutralizing antibody to eCIRP. Additionally, markers for organ damage such as aspartate transaminase (AST) and alanine transaminase (ALT) were also elevated by the presence of eCIRP and significantly reduced upon its inhibition with the antibody. The effects of eCIRP-neutralizing antibody were again demonstrated in hemorrhaged rats (*n* = 6) using the method described above. The administration of anti-CIRP to hemorrhaged rats significantly reduced serum and hepatic levels of TNF-α and IL-6. The pro-inflammatory state and resultant organ damage contributed to mortality after shock as shown by the increased survival rate of CIRP knockout (*CIRP*^−/−^) mice 72 h after hemorrhage (*n* = 9), with 56% survival for the knockout group vs. 11% survival for the wild-type group. Furthermore, myeloperoxidase activity in the liver was also reduced in the *CIRP*^−/−^ mice, suggesting an interplay between eCIRP and neutrophil activity [15]. In our more recent studies, we have transitioned to a mouse model for hemorrhagic shock. The procedure is somewhat similar across various institutions. Briefly, in our lab, bilateral femoral arteries are cannulated with one side for bleeding and the other to monitor and sustain blood pressure at a level coinciding with shock. For mice, MAPs are maintained from 25 to 30 mm Hg for 90 min. Resuscitation of two times the shed blood volume using Lactated Ringer’s solution is then administered over a period of 60 min. Other models of hemorrhagic shock include bleeding from retro-orbital puncture or carotid artery cannulation. Resuscitation is achieved via venous or arterial approach. The most highly used model is described in detail in this referenced study [46].

### 3.2. eCIRP and Hepatic Ischemia/Reperfusion (I/R)

As previously discussed, one of the hallmarks of hemorrhagic shock is organ ischemia and subsequent reperfusion injury following resuscitation. eCIRP has also been implicated in the development of I/R injury in both the liver and kidneys. In the liver, ischemia–reperfusion was simulated in mice by clamping the hilum of the left and medial liver lobes, resulting in 70% ischemia. The ischemic state was maintained for 60 min followed by reperfusion by releasing the vessel clamps. Serum from the hepatic I/R and sham groups (*n* = 3–4) were then harvested at various timepoints after reperfusion (1 h, 4 h, and 24 h). eCIRP levels in circulation were noted to be slightly increased at 4 h and increased up to 6-fold from control mice at 24 h post-reperfusion. Serum markers for liver damage including AST, ALT, and lactate dehydrogenase (LDH) were also increased after I/R; however, the increase was ameliorated when eCIRP activity was blocked using anti-CIRP antibody. Mice that were treated with anti-CIRP antibody (*n* = 6) had attenuated liver injury and systemic inflammatory response with decreased serum inflammatory cytokine levels, reduced neutrophil infiltration rate within harvested liver, and decreased apoptosis and oxidative stress. Liver injury was quantified using histologic liver injury score, which is the total sum of five different parameters: cytoplasmic color fading, cellular vacuolization, sinusoidal congestion, necrosis, and erythrocyte stasis. The 10-day survival rates of mice with reperfusion injury significantly increased when treated with anti-CIRP antibody (*n* = 16), with a 37.5% survival rate for the I/R group vs. a 75% survival rate for the anti-CIRP treated group [47]. The mouse model involving partial ischemia of the left and medial lobes has been in use across various institutions. The steps of the procedure were defined by Dr. Grisham’s group in 2009 [48].

### 3.3. eCIRP and Renal I/R

Similar findings were demonstrated in renal ischemia–reperfusion injury. Using a mouse model, renal ischemia was induced via bilateral clamping of the renal pedicles for 30 min followed by reperfusion for 24 h. Bilateral kidneys were accessed through a midline laparotomy. This technique is favored by the majority of other study authors; however, alternatives include right nephrectomy to collect sham samples plus left renal vessel clamp or the use of a flank incision to access one kidney. The bilateral clamping method has recently been validated in both mice and rats [49]. Renal tissue was then harvested at either 5 h or 24 h post-I/R injury and compared to renal tissue harvested from sham mice who underwent midline laparotomy without I/R (*n* = 5–9). Like the liver, CIRP expression in the kidneys was increased after reperfusion, with a 4-fold increase at 24 h [50]. Serum BUN and creatinine levels were measured to indicate the level of acute kidney injury from reperfusion. BUN and creatinine levels were significantly reduced in *CIRP^−/−^* mice (*n* = 5–6) compared to wild-type mice (*n* = 3–4), suggesting the involvement of eCIRP in acute kidney injury. Randomized and blinded histological analysis of 10 randomly selected fields also demonstrated decreased tubular cell injury, tubular cell detachment, the loss of brush border, tubular simplification, and cast formation in *CIRP^−/−^* mice [50]. The destruction of tubular cells is a hallmark of acute kidney injury and acute tubular necrosis, both of which occur frequently in trauma patients [51]. Similar to the liver, mice undergoing renal I/R had decreased levels of IL-6, caspase-3, neutrophil infiltration, and oxidative stress when anti-CIRP antibody treatment was administered [50]. The decrease in oxidative stress was demonstrated via the decreased expression of an oxidatively modified protein marker, nitrotyrosine, and the decreased mRNA expression of *cyclooxygenase-2* (*COX-2*) in *CIRP^−/−^* mice subjected to renal I/R (*n* = 4–7) vs. sham mice (*n* = 3–4). The effects of CIRP on kidney damage were further demonstrated by comparing BUN levels in the serum of mice treated with and without anti-eCIRP antibody (*n* = 5–9 per group). BUN levels were significantly reduced by 81% when eCIRP antibody was administered.

These studies underscore the involvement of eCIRP in hemorrhagic shock and related sterile inflammation conditions such as ischemia–reperfusion injury. By understanding its involvement in I/R injury, we can further study the nature and development of multi-organ failure. Additionally, hemorrhagic shock in the setting of trauma is rarely isolated to blood loss and is usually accompanied by polytraumatic injuries. Our data suggest eCIRP is involved in both systemic and local inflammation, which can be a key benefit in treating polytrauma. eCIRP’s functions, as a DAMP in multiple aspects of shock, including hypoperfusion, organ injury, and SIRS highlight its potential as a therapeutic agent in resuscitation and as a targeted drug therapy.

### 3.4. eCIRP and Other Human Pathologies

Although the above studies regarding eCIRP in hemorrhagic shock demonstrated statistically significant results, more details regarding the preclinical survival studies would be beneficial in the transition to clinical studies. To the best of our knowledge there are no current therapeutics targeting DAMPs that are approved as treatment for hemorrhagic shock. At this time, eCIRP studies in hemorrhagic shock are mostly limited to the preclinical stage except for the 10 patients reported in our 2013 *Nat Med.* article [15]. Since our discovery of eCIRP as a DAMP, several articles have described the association of eCIRP to poor outcomes in sterile inflammation induced by blood loss in other human pathologies. eCIRP is associated with the increased risk of cardiac surgery-associated acute kidney injury (AKI). When comparing serum pre- and post-operatively from 292 individuals who underwent cardiac surgery, a greater increase in eCIRP was independently associated with AKI. Among the total 292 patients, 118 experienced AKI after the surgery. A total of 249 patients among the total also underwent cardiopulmonary bypass during the surgery. Increased cardiopulmonary bypass times were also correlated with increased levels of eCIRP [52]. In another study, where 96 patients undergoing total aortic arch replacement under hypothermic circulatory arrest after a Stanford A dissection, eCIRP levels increased at 12 h post-operation and peaked at 24 h. The higher levels of eCIRP was deemed an independent risk factor for post-operative 30-day mortality [53]. Furthermore, a total of 73 patients who underwent cardiac arrest with subsequent spontaneous return of circulation (ROSC) were divided into non-survivor (*n* = 48) and survivor (*n* = 25) groups based on 28-day survival. eCIRP levels were measured on days 1, 3, and 7 after ROSC. eCIRP levels were significantly increased within the first week of ROSC. eCIRP was positively correlated with an increased systemic inflammatory response, increased Sequential Organ Failure Assessment (SOFA), and Acute Physiology and Chronic Health Evaluation (APACHE II) scores. eCIRP levels on days 1, 3 and 7 after ROSC were predictive of 28-day mortality and neurological prognosis [54]. While there is no substantial amount of clinical data on eCIRP and trauma hemorrhage, the above studies mimic the state of hemorrhagic shock, albeit in a controlled situation. Cardiac arrest is the eventual outcome of untreated shock, and the data can be similarly applied to hemorrhagic shock. Based on these clinical findings, eCIRP may serve well as a diagnostic tool for patients admitted with hemorrhagic shock and help guide clinical decision making.

## 4. eCIRP’s Mechanisms of Action

The studies discussed above implicate eCIRP in the generation of tissue damage via increased cytokine secretion, apoptosis, and neutrophil activity. Its function as a DAMP necessitates binding with an affiliated PRR. eCIRP can exert its deleterious effects by producing local tissue damage at the time of injury as well as post-resuscitation. Its mechanisms at these differing time points as well as the cell types and locations exert maximal effect in all areas of interest. In this section, we aim to further detail the inflammatory cascade promoted by eCIRP in hemorrhagic shock.

### 4.1. TLR4 as an eCIRP Receptor

In hemorrhagic shock, toll-like receptor-4 (TLR4) has been identified as a crucial inflammatory mediator receptor, resulting in increased organ damage, coagulopathy, and the physiological regulation of vascular tone. TLRs, initially discovered in Drosophila, were the first family of PRRs to be characterized [55]. TLRs comprise the toll subgroup of the interleukin-1 receptor/toll-like receptor superfamily which contain the toll-interleukin-1 receptor (TIR) homology domain [56,57]. TIR domains, intracellular signaling domains responsible for activating further downstream signaling to induce inflammation and programmed cell death, are evolutionarily conserved [58]. TLRs are type I transmembrane glycoproteins composed of an extracellular region, transmembrane region, and intracellular region. In humans, 10 members of the TLR family have been identified, and TLR4 was the first homolog discovered. They share a common structural motif of leucine-rich repeats (LRR), each containing 20–30 amino acids located on the horseshoe-shaped N-terminus [59]. The extracellular LRR identifies PAMPs and DAMPs, while the intracellular TIR domain recruits adaptor molecules such as MyD88, TIRAP, TRIF, and TRAM. The final downstream effect of this interplay results in the activation of NF-kB and the release of TNF-α, IL-1β, IL-6, and chemokines. TRAM, which is exclusive to TLR4, also activates IRF3, and releases IFN-β, MCP-5, RANTES, and nitric oxide [60].

**Figure 1:**
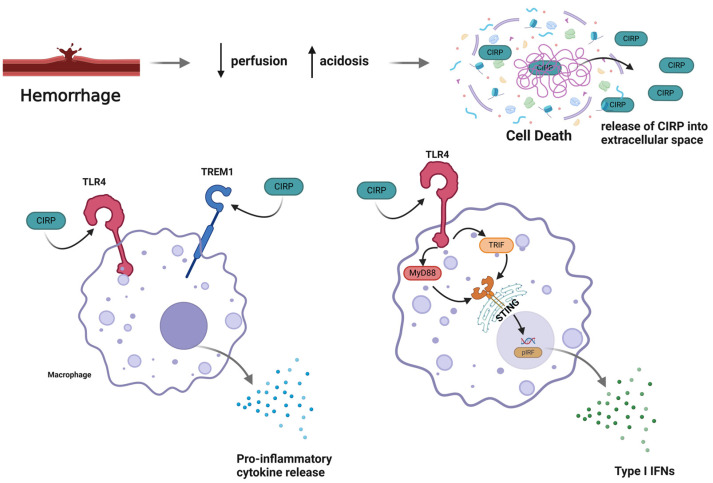
Mechanisms of action of eCIRP in hemorrhagic shock. Damaged vessels are unable to deliver blood to tissue due to hemorrhage. The loss of perfusion and resultant hypoxemia result in ischemic damage to cells. Due to hypoxemia, cellular metabolism shifts to anaerobic metabolism via glycolysis which leads to increasing lactate production. The accumulation of lactate creates an acidotic environment, causing further cell damage. The accumulation of these factors results in cell death by varying mechanisms including apoptosis, necrosis, and necroptosis. As a result of cell death, the cell membrane is disrupted, and intracellular CIRP is released into the extracellular space, becoming eCIRP, as shown in the top right. During the shock state, blood flow is shunted towards vital organs. eCIRP, now in systemic circulation, will also be shunted towards these organs, causing increased inflammation. eCIRP will bind to TLR4 and triggering receptor expressed on myeloid cells (TREM1) receptors on both tissue and circulating macrophages, demonstrated in the bottom left. As a result of this binding, the macrophage will release pro-inflammatory cytokines into circulation, worsening inflammation in the already ischemic tissues. A detailed image shown in the bottom right illustrates eCIRP acting on TLR4/MyD88 and inducing damage to the mitochondria, causing an increase in cytosolic DNA. This cytosolic DNA via the cGAS pathway activates stimulator of interferon genes (STING) on the endoplasmic reticulum membrane. This results in an increase in pIRF, a transcription factor responsible for increasing expression of type I *IFNs*. The activation of this pathway increases the release of type I IFNs into the circulation. These pathways collectively lead to inflammation and injury in hemorrhagic shock.

In vivo experiments have demonstrated increased cell surface TLR4 in resuscitated hemorrhagic shock-treated rats with an increase of >38% compared to wild type [61]. On both dendritic cells (DC) and myeloid cells, the deletion of *TLR4* gene leads to the near complete inhibition of systemic cytokine and chemokine released following hemorrhagic shock. Markers of inflammation, like IL-6, and markers for end-organ damage, like ALT, were significantly increased in mice with *TLR4^+/+^* DC but not with *TLR4^−/−^* DC [62]. TLR4 has also been implicated in the inflammatory response mediated by eCIRP in hemorrhagic shock. In the previously cited study regarding CIRP’s role in hemorrhagic shock and sepsis, knockout mice targeting three major PRRs (*RAGE*, *TLR2*, and *TLR4*) were tested for TNF-α response to recombinant CIRP. The loss of the response was only exhibited in *TLR4*-deficient mice. Similarly, the increase in serum levels of TNF-α, IL-6, and HMGB1 after recombinant CIRP injection was not observed in *TLR4^−/−^* mice [15]. Myeloid differentiation factor 2 (MD-2) is a molecule that is found in association with TLR4 and was first discovered as a necessary co-receptor for TLR4 to mount a response against LPS from pathogens [63]. CIRP amino acid segment 106–125 was noted to also have a high binding affinity (K_d_ 3.02 × 10^−7^ M) when MD2 was part of the TLR4/MD2 complex [15]. This evidence strongly indicates that eCIRP’s mechanisms of action are mediated at least in part by the TLR4 signaling pathway (Figure 1).

### 4.2. TREM-1 as Another eCIRP Receptor

The triggering receptor expressed on myeloid cells/DNAX activation protein 12 (TREM1)/DAP12) pathway is also involved in the systemic inflammatory response and organ dysfunction found in hemorrhagic shock. TREM-1 is an immunoglobulin (Ig) superfamily transmembrane receptor expressed on the surface of myeloid cells such as granulocytes, monocytes, and tissue macrophages [64]. They are expressed by the TREM gene on chromosome 6p21.1. Like TLRs, they are evolutionarily conserved among species with *TREM1*, *TREM2*, *TREML1*, *TREML2*, and *NCR2* representing the human variants [65]. The activation of the TREM-1 receptor results in the production of reactive oxygen species, pro-inflammatory cytokines, and chemokines, as well as the degranulation of neutrophils and phagocytosis via the DAP12-mediated signaling pathway. DAP12 is a 12kDa protein which contains a cytoplasmic immunoreceptor tyrosine-based activation motif (ITAM) responsible for linking the extracellular activation of TREM1 to the intracellular signaling pathways involved in inflammation [66]. The TREM1/DAP12 pathway can also synergize with TLR4, thus further amplifying the innate immune response [67]. In response to TLR4 activation, *TREM-1* expression is also up-regulated. As a result, the production of pro-inflammatory cytokines such as TNF-α and GM-CSF are increased, while the anti-inflammatory cytokine IL-10 is inhibited [68].

Arterial blood gas drawn from hemorrhagic shock mice treated with TREM1 decoy receptor LP17 had decreased lactate levels. After resuscitation, lactate levels continued to decrease in the LP17 group, while they plateaued in the control group. Blocking the effects of TREM1 using LP17 during resuscitation attenuated lactic acidosis and the degree of shock found in these mice. AST levels and urea concentration were decreased by LP17, suggesting a protective role in liver and kidney dysfunction. In the lung, the loss of pulmonary alveolar endothelial and epithelial integrity was mitigated when bypassing the TREM1 pathway [69]. AKI is a common complication with an incidence of 42% in patients presenting with hemorrhagic shock [70]. Serum BUN and creatinine levels were significantly lower in *TREM-1^−/−^* mice. Other markers for AKI and inflammation such as kidney injury molecule-1 (KIM-1) and neutrophil gelatinase associated lipocalin (NGAL) were comparatively reduced in the *TREM-1^−/−^* mice. eCIRP can activate TREM-1, leading to its downstream inflammatory effects. In the kidney, AKI induced via the injection of recombinant CIRP had a decreased effect in *TREM-1^−/−^* mice [71]. TREM-1 also plays a role in eCIRP-induced hepatic I/R injury. Both *TREM-1* gene expression and eCIRP are markedly elevated after hepatic I/R induction. Using the method of clamping the hilum of left and medial liver lobes to achieve 70% ischemia, wild-type and *TREM-1^−/−^* mice were compared. The degree of histological damage along with levels of AST, ALT, and lactate dehydrogenase (LDH) were decreased in *TREM1^−/−^* mice. In the absence of *TREM-1*, local and systemic inflammatory response is dampened. Myeloperoxidase (MPO) activity, used to determine the degree of neutrophil infiltration [72], was decreased along with chemokine macrophage inflammatory protein-2 (MIP-2) and neutrophil inflammatory marker COX-2 [73]. When the interaction between eCIRP and TREM-1 was inhibited using a novel peptide inhibitor, M3, discussed later in this article, these inflammatory changes were all attenuated. These observations indicate that in addition to the TLR4 signaling pathway, eCIRP’s inflammatory effects are mediated by the TREM-1 signaling pathway (Figure 1).

### 4.3. eCIRP and STING

Another mechanism by which eCIRP induces inflammatory effects during hemorrhagic shock is through STING (Stimulator of interferon genes) activation. STING, also known as transmembrane protein 173 (TMEM173), is an activator of the innate immune response. STING is located on the membrane of the endoplasmic reticulum (ER), consisting of 379 amino acids with its C-terminal extending into the cytoplasm [74]. As an intracellular recognition receptor, its role is to modulate transcription via the cGAS-STING signal pathway. When cGAS, an intracellular nucleic acid sensor, detects double-stranded DNA (ds-DNA) in the cytosol, it will catalyze the production of cGAMP which then binds to the C-terminal of STING on the ER membrane [75,76]. Subsequently, the STING molecule translocates to the Golgi apparatus to be packaged into perinuclear microsomes which then activate transcription of NF-kB, TANK-binding kinase 1 (TBK1), and transcription factor interferon regulatory factor (IRF3). The endpoint of this cascade is the activation of type 1 interferons (IFNs) [74,76]. These cytokines stimulate macrophages, natural killer cells, and also recruit CD8+ cytotoxic T-cells via classic antigen-presenting dendritic cells [77].

One mechanism by which Type I IFN can induce its inflammatory effects during HS is through eCIRP-induced STING activation ([78]; Figure 1). Hemorrhagic shock was induced in mice through the bilateral cannulation of femoral arteries to maintain a MAP of 27 +/− 2.5 mmHg for 90 min. Animals were then resuscitated with lactated Ringer’s solution for 30 min. Blood and lungs were collected 4 h post-resuscitation. *STING^−/−^* mice had significantly decreased serum ALT, AST, IL-6, and IFN-β compared to wild-type mice. In the lungs, the mRNA expression of *CCL3*, *CCL8*, and *CXCL* was significantly reduced in addition to the expression of *COX2* and *iNOS* mRNA in *STING^−/−^* mice. The deficiency of *STING* also led to decreased MPO activity and improved lung injury scores, suggesting an overall protective effect against acute lung injury (ALI). The injection of recombinant CIRP increased the levels of downstream STING pathway targets, pTBK1 and pIRF3, leading to the increased expression of *IFN-α* and *IFN-β* mRNA in the lungs.

The interplay between STING and eCIRP involves both the TLR4-MyD88 signaling pathway and fragmented mitochondrial DNA. Under normal conditions, STING is activated by cytosolic DNA. During stress conditions, the released eCIRP acts on the TLR4-MyD88 signaling, results in the fragmentation of mitochondrial DNA. The fragmented mitochondrial DNA is then localized to the cytoplasm and activates STING, leading to IFN production. This subsequent increase in IFNs is almost completely inhibited in *TLR4^−/−^* and *MyD88^−/−^* macrophages [78]. Toll-IL-1 receptor domain-containing adaptor inducing IFN-β (TRIF) signaling is another signaling pathway involving the TLR family. TRIF leads to dendritic cell maturation and serves as the link to the adaptive immune system [79]. eCIRP-treated macrophages had a more pronounced effect on *IFN-α* and *IFN-β* expression in wild-type mice compared to *TRIF^−/−^* mice [78]. H151 is a potent, selectively binding, and irreversible inhibitor of STING developed by Invivogen [80]. Its binding site on STING is located at the transmembrane cysteine residue at position 91. As a result, STING activation is blocked, and the downstream IFN production is strongly reduced. In the context of AKI, H151 exerts its effect by inhibiting eCIRP-induced STING activation. Renal I/R-treated mice had diminished levels of IL-6, TNF-α, and NGAL when co-treated with H151 [81].

These studies indicate that both TLR4 and TREM-1 act as receptors for eCIRP to induce the inflammatory response in hemorrhagic shock and other related injury conditions (Figure 1). In addition, eCIRP-induced STING activation leading to type I IFNs has been implicated as a potential eCIRP mechanism (Figure 1). From our current knowledge eCIRP exerts its effect via macrophages. As a DAMP that enters circulation, it would also be beneficial to understand the role of eCIRP in other HS-associated processes [82]. We have not completely resolved how eCIRP induces endothelial and epithelial damage, coagulopathy, and alters neutrophil function, all of which are complications associated with organ dysfunction in hemorrhagic shock.

## 5. eCIRP as a Therapeutic Target for Hemorrhagic Shock

**Figure 2:**
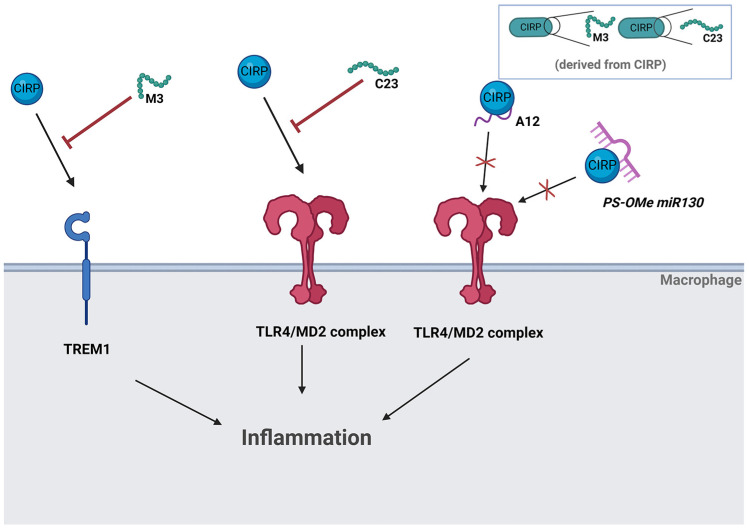
eCIRP as a therapeutic target in hemorrhagic shock. Both C23 and M3 are small peptides derived from the human sequence of CIRP. C23 has high affinity to the TLR4/MD2 complex, and M3 has specific binding for the TREM1 receptor. Thus, both peptides block the binding of eCIRP to their respective receptors. In contrast, A12 is a synthetic oligonucleotide consisting of a poly(A) tail mimic that binds to eCIRP, blocking its binding site to the TLR4/MD2 complex. PS-OME miR 130 is a microRNA with stabilizing adjustments, including 3 phosphorothioate (PS) bonds at the 5′ and 3′ ends and 2′Omethyl (2′Ome) bases. It has a strong binding affinity to eCIRP, thereby preventing eCIRP from signaling via the TLR4/MD2 signaling pathway. As a result, these small molecule peptides and oligonucleotides prevent the interaction between endogenous eCIRP and its target receptors on macrophages. As a result, the inflammatory cascade is inhibited, and the hyperinflammatory response at the initial insult from hemorrhagic shock is prevented.

As the mechanisms by which eCIRP act on hemorrhagic shock-associated inflammation are further elucidated, novel targets to modulate these pathways are of increasing interest. Polytrauma patients have a long period of recovery and are at high risk during their hospital stay. Blunt trauma, especially, is difficult to treat, and therapy is generally limited to observation and resuscitation. The combination of hypoperfusion from shock as well as the injuries sustained from polytrauma generate an overactive inflammatory cascade. Currently, we have limited options on mitigating this effect. Thus, it is crucial that we develop therapies that can reduce the hyperinflammatory state after shock, thereby shortening the recovery period and improving outcomes (Figure 2).

### 5.1. C23

C23 is a short (15-mer) peptide derived from human CIRP that binds to the TLR4/MD2 complex. Using surface plasmon resonance (SPR), this peptide was identified to have the highest affinity and a binding site within the MD2 pocket. Thus, it acts as a competitive inhibitor to reduce the inflammatory effects of eCIRP. In an in vitro model, C23 administration led to a dose-dependent reduction in TNF-α production in macrophages stimulated with eCIRP. In vivo, kidney inflammation following kidney I/R is attenuated in C23-treated mice. Local *TNF-α* expression was significantly reduced, and the average injury score under light microscopy examination was reduced in C23-treated mice compared to vehicle-treated mice. Specific AKI markers, NGAL and KIM-1, were reduced by C23, and apoptosis decreased significantly in TUNEL assay. Impressively, overall survival rate 8 days after renal I/R induction increased when treated with C23 [83]. Studies in the lung showed similar findings. Trauma-induced acute lung injury has an incidence of 30–50% [84]. Acute lung injury can progress to acute respiratory distress syndrome which has a high mortality rate of 34–58% [85]. As hemorrhagic shock often leads to respiratory failure, lung tissue from mice was assessed for inflammatory changes. The assessment of lung tissue in C23-treated mice revealed decreased neutrophil infiltration and expression of pro-inflammatory cytokines *IL-1β*, *TNF-α*, and *IL-6*. Lung histological injury score was improved with C23 treatment. Like the kidney, endothelial cells in the lung had improved function with decreased expression of *ICAM-1* and decreased vascular permeability. The decrease in vascular permeability led to a reduction in edema and reduced sequestration of neutrophil debris [86]. The significant improvement in biochemical parameters and survival illustrates the potential of C23 as a novel therapy in both renal I/R injury and trauma-induced acute lung injury. Of note, C23 targets TLR4 and TLR4 antagonists have failed in prior clinical trials, therefore the potential to develop C23 as a therapeutic in humans could prove to be challenging.

### 5.2. M3

M3 is a newly developed, novel 7-amino acid peptide derived from human CIRP sequence. It acts as an inhibitor between eCIRP and TREM-1, resulting in the inhibition of TNF-α and IL-6 production by macrophages [87]. In hemorrhagic shock, M3 treatment reduced serum levels of IL-6 and TNF-α. As with C23, renal and lung injury was assessed using M3 treated mice. The examination of lung tissue revealed significant decreases in *IL-6*, *TNF-α*, and *IL-1β* expression. mRNA expression of the chemokines, *MIP-2*, and *KC* in the lung were also significantly reduced. Histological lung injury score was improved by 52% [88]. M3 demonstrated a protective effect against AKI after renal I/R. The activation of renal endothelial cells via eCIRP-TREM-1 binding results in worsened AKI. Human renal glomerular endothelial cells (HRGECs) exhibited increased secretion of VCAM-1, IL-2, IL-6 and chemoattractants SCXCL1, CCXL5, MCP-1, IL-8, as well as PDGF-AA and proangiogenic factors including osteopontin and angiogenin. This cascade is mitigated via M3-induced inhibition. Damage to renal architecture was less severe and the number of apoptotic cells decreased significantly. There was also an overall survival benefit demonstrated in the 10-day survival rate in M3-treated renal I/R mice [71,89]. Similar results were replicated in hepatic I/R mice. Local inflammation was markedly reduced with M3 treatment. Upon administration of M3, there was a significant decrease in neutrophil infiltration demonstrated by MPO levels along with a significant decrease in *IL-6* mRNA expression. Other local inflammatory markers such as *MIP-2* and *COX-2* were reduced with M3 treatment as well. 10-day survival benefit was also observed in hepatic I/R mice treated with M3 [73]. As M3 is a peptide and since it targets TREM1, rather than TLR4, it has some potential for translation to clinical trials. Peptide therapeutics are currently in use for type II diabetes mellitus, cardiovascular disease, gastrointestinal disease, and cancer [90]. To avoid detrimental side effects, specificity would need to be ensured so that the immune system continues to be active against infectious agents during the patient’s recovery period.

### 5.3. A12

A12 is a synthetic oligonucleotide consisting of a poly(A) tail mimic comprising 12 adenosines which is further modified with 2′-O-methyl ribose and terminal phosphorothioate links. The modifications provide protection against RNases degradation, thereby increasing its half-life. A12 shows a strong affinity for eCIRP and competitively inhibits the binding of eCIRP to TLR4, effectively inhibiting both systemic and local inflammation [91]. These findings were initially revealed in sepsis but have since been replicated in hemorrhagic shock. Treatment with A12 in hemorrhaged mice significantly reduced serum IL-6 and TNF-α when compared to the vehicle group. When analyzing lung tissue specifically, beneficial effects of A12 were further demonstrated. Harvested lung tissue from the hemorrhaged mice contained a significant reduction in tissue *IL-6* and *TNF-α* expressions. *MIP-2* and *KC*, markers for neutrophil infiltration, were significantly reduced with A12 treatment. Histological injury score, MPO activity, and apoptosis levels in TUNEL assay were reduced, collectively indicating its pro-therapeutic role in hemorrhage-induced acute lung injury. A12 did not exhibit any cytotoxic effect either, as there was no difference in inflammatory cytokine levels between A12-treated and sham groups. The lack of cytotoxic effect coupled with significant anti-inflammatory benefits demonstrates the therapeutic potential of A12 in hemorrhagic shock-induced lung injury [92]. However, drug delivery of oligonucleotides continues to be a challenge, and few agents have passed into the clinical phase with FDA approval. Hemorrhagic shock is a systemic process, and systemically delivered oligonucleotides are at risk of degradation in the extracellular space. Additionally, oligonucleotides undergo rapid renal clearance, and when coupled with the increased inflammatory state in shock, the risk of failed drug delivery runs higher [93]. Nonetheless, as drug delivery methods continue to advance, A12 with its high affinity for eCIRP remains a promising target. Since eCIRP not only recognizes TLR4 but also several other receptors, such as TREM-1 and IL-6 receptor, its binding to receptors other than TLR4 can be prevented by binding A12 instead. This is a highly advantageous feature in preventing eCIRP-induced inflammation and injury in hemorrhagic shock. Although there is high specificity of A12 to eCIRP, one cannot rule out the possibility that A12 could have off-target effects. A more comprehensive approach is required to fully delineate the interaction of A12 with other ligands to provide a broader understanding of the effect of A12 in inflammatory conditions including hemorrhagic shock.

### 5.4. PS-OME miR130

Circulating RNA products such as microRNA exert an effect on eCIRP. miRNA 130b-3p is a microRNA that inhibits eCIRP-mediated inflammation. This finding was first elucidated using sepsis models [88]. As miRNA is highly degradable, PS-OMe miR130 was developed with stabilizing adjustments including 3 phosphorothioate (PS) bonds at the 5′ and 3′ ends and 2 O’methyl (2′Ome) bases scattered throughout [94,95,96]. This miRNA 130 mimic was thus evaluated in hepatic and renal I/R. In hepatic I/R, treatment with PS-OMe miR130 led to significantly decreased AST, ALT, and LDH. Liver histology injury score was markedly improved. Local inflammation was significantly reduced as evidenced by decreased *TNF-α* and *IL-1β* mRNA expression as well as decreased MPO activity. Apoptosis, as well, was markedly reduced with PS-OMe miR130 treatment. For renal I/R, PS-OMe miR130 treatment showed a significant decrease in organ injury markers BUN, NGAL, KIM-1, and inflammatory cytokine, IL-6. Gr-1 immunohistochemistry, used to examine the degree of neutrophil infiltration, was significantly reduced, and average histological injury score was also decreased. TUNEL assay demonstrated a significant decrease in apoptosis with PS-OMe miR130 treatment. The 10-day survival was also improved in renal I/R mice with PS-OMe miR130 treatment [97]. Small molecules have been favored as leading candidate for drug development because of their ability to interact with biological targets within intracellular and extracellular spaces. However, their versatility is coupled with off-target effects. Although PS-OMe miR130 is modified to increase stability, much like A12, similar issues of off-targeting can occur with microRNA therapy. While we have not unveiled any conspicuous adverse effects, given the potential adverse effects with alterations in dosage and time point, more judicious examination is warranted for the safety of the molecule for therapy.

### 5.5. Considerations for eCIRP as a Therapeutic Target in Hemorrhagic Shock

When considering the treatment of hemorrhagic shock, one must consider both the time of injury, control of bleeding, and subsequent management during the observational period. The studies discussed implicate eCIRP as a promoter of the hyperinflammatory response during shock. Although pro-inflammatory cytokines are necessary to clear necrotic tissue caused by ischemia, during the reperfusion stage, these cytokines inadvertently cause more damage. By dampening this response, the amount of organ damage can be mitigated, thus preventing organ failure, multiple organ failure, and death. Current hemorrhagic shock treatment focuses strongly on early resuscitation. Resuscitation with blood products, while improving circulation and preventing ischemia, also comes with the risk of reperfusion injury. Thus, we believe anti-eCIRP agents may be a strong candidate as a therapeutic at the time of resuscitation. Patients presenting to the trauma bay or operating room in hemorrhagic shock are unable to receive medications through the oral route. To be effective at this time point, anti-eCIRP therapies would ideally need to be available as an IV solution or injection. This provides its own set of difficulties, as the therapeutics discussed above are highly degradable. Additionally, blood products are not always readily available in the field, especially in rural areas or in military combat regions. In these scenarios, the amount of space is a concern. Although they cannot replace resuscitation or hemorrhage control, the smaller size of molecular inhibitors is another benefit during transport and early treatment. By administering these agents early, the deleterious effects of prolonged shock can be mitigated. Another point of consideration is ongoing hemorrhage and drug availability within the bloodstream. If anti-eCIRP agents are delivered directly into the bloodstream while hemorrhage is ongoing, it is likely that much of the product will be lost from continued blood loss. Surgical and endovascular treatment interventions remain the mainstay of controlling blood loss. Anti-eCIRP can be a strong adjunct to treatment once hemorrhage is controlled and reperfusion begins. Lastly, it is also important to consider the molecular processes that occur during hospital admission. eCIRP levels do not peak immediately and continue to rise days after the initial traumatic insult. Although further clinical data are required to make a definitive statement on its actual peak levels in humans, the data we have available currently point towards elevations in eCIRP levels at least 1–3 days after injury. Elevated levels of eCIRP, along with other DAMPs, are associated with worse outcomes. As a diagnostic marker, eCIRP can give clinicians a better understanding of the underlying damage as patients recover from traumatic injury and hemorrhage. Patients with elevated levels of eCIRP in the bloodstream are more likely to develop organ failure [49]. Anti-eCIRP therapies can be tailored to suit the needs of varying patients and can provide a more targeted approach as well. The continued monitoring of eCIRP along with anti-eCIRP therapeutics, as needed, would help to prevent mortality. As we have no clinical trials for anti-eCIRP as a therapy for humans in general, it is difficult to say how these drugs would fare when administered orally. For optimal results, these therapies would need to withstand degradation in the gastrointestinal system. Further research is needed for a better understanding on its metabolism as well. It is currently unknown whether these agents are cleared by the liver or kidney. As is the case with any other drug, careful consideration must be made regarding drug toxicity, as post-shock patients are in a hypermetabolic as well as immunosuppressed state.

## 6. Future Studies and Perspectives

Hemorrhagic shock continues to be a significant source of mortality worldwide. Even after the initial insult, patients are susceptible to several morbid, potentially fatal, complications including the risk of developing a single organ or multiple organ failure. Research focusing on the innate immune response has helped us gain a greater understanding of the overactive inflammatory cascade in hemorrhagic shock. Our current research has a strong focus on eCIRP and its associated receptors. At this time, there are no clinical trials studying the effects of eCIRP in traumatic hemorrhagic shock. The mechanisms discussed above are promising targets for therapeutic intervention. Ongoing studies aim to develop and further advance novel agents that have potential applications clinically. As our insight into the field progresses, the next steps would be translation into the clinical sector, with the hopes of developing new drugs aimed at preventing organ damage and failure. To develop these molecules as therapeutics for HS in humans, additional preclinical work including therapeutic window, optimal administration route, and optimal dose for administration is needed. Continued in vivo work with varying time points of drug administration along with studies testing drug metabolism are among the next steps. In addition, although these molecules are peptides and less likely to be toxic in vivo, additional short-term and long-term toxicity profiles; pharmacokinetic analysis including absorption, distribution, and metabolism; and excretion (ADME) studies are needed. Challenges to the translational phase also include drug delivery methods and the current lack of human data. Further studies using human samples and human-derived cell lines are needed before we can consider moving into the translational or clinical phase. Clinical studies, both prospective and retrospective, with larger sample sizes are needed to validate the currently available data. There is also a lack of systemic meta-analyses regarding molecular medicine, hemorrhagic shock, trauma and mortality. The biggest challenge in traumatic hemorrhagic shock is that the nature of injury is multifactorial. Analysis on the cause of mortality is crucial so that investigators can tailor the development of different therapies. Additionally, further research is needed on immunosuppression, which is a key feature of late-stage complications. Although the goal of eCIRP, along with other DAMP blockade, is to prevent the overactive inflammatory cascade, it is imperative that the immune system continues to function well against secondary infections during hospitalization. The SIRS response, caused by the hyperinflammatory state from DAMPs, continues to be a challenge in trauma care. With mortality rates reaching 40% and 28-day mortality rates at 20% or more, it is imperative that we improve our understanding of the physiological and molecular processes that occur from shock so that we may reduce disease burden and improve the outcomes for victims of trauma.

## Data Availability

Not applicable.
